# Ruptured intra-abdominal testicular seminoma with hemorrhage shock, after inadequate surgical exploration for undescended testis: a case report

**DOI:** 10.1186/s40792-021-01143-5

**Published:** 2021-03-08

**Authors:** Hirotake Gonda, Takuya Saito, Takaaki Osawa, Shintaro Kurahashi, Tatsuki Matsumura, Yasuyuki Fukami, Shunichiro Komatsu, Kenitiro Kaneko, Kazuhiro Hiramatsu, Takehito Kato, Tsuyoshi Sano

**Affiliations:** 1 Division of Gastroenterological Surgery, Department of Surgery, Aichi Medical University, 1-1 Yazakokarimata, Nagakute, Aichi 480-1195 Japan; 2 Department of Surgery, Toyohashi Municipal Hospital, Toyohashi, Aichi Japan

**Keywords:** Undescended testis, Seminoma, Intra-abdominal hemorrhage

## Abstract

**Background:**

Undescended testes are associated with an increased risk of malignancy and infertility, and surgical treatment in childhood is recommended.

**Case presentation:**

A 35-year-old man presented to the emergency department with abdominal pain and vomiting. Despite a history of surgery for a left undescended testis in infancy, his left-sided scrotum appeared underdeveloped. Contrast-enhanced computed tomography showed a pelvic mass, involving a major axis of approximately 15 cm, with high-density ascites suggestive of hemorrhage. A ruptured gastrointestinal stromal tumor was suspected. As he was in hemorrhagic shock, an emergency laparotomy was indicated. The active bleeding mass was controlled through complete resection. A pathological evaluation of the mass revealed a seminoma arising from an undescended testis. His post-operative course was uneventful, and he was discharged on post-operative day 6. Recurrence on the retroperitoneal lymph nodes was detected 1 year postoperatively, and a retroperitoneal lymph node dissection was performed after chemotherapy. He remains well without any apparent signs of recurrence.

**Conclusions:**

Paying close attention to an empty scrotum is advisable, even postoperatively, for undescended testis because of possible subsequent potential malignancy presenting with hemorrhage, as our patient demonstrated.

## Background

Undescended testes are a common birth anomaly [1]. Approximately 10% of undescended testes are located in the abdominal cavity, and an intra-abdominal testis has been reported to be at a higher risk of testicular cancer than an inguinal testis [2, 3]. Orchiopexy is recommended at approximately 12 months of age to reduce the risk of testicular tumors and infertility [1, 2]. However, undescended testes potentially remain in the abdominal cavity, even after orchiopexy, for various reasons [4]. Here, we report a patient with intra-abdominal hemorrhage from a left testicular seminoma in the abdomen. Although there are many reports of intra-abdominal testicular seminoma, presentation with hemorrhagic shock due to tumor rupture is extremely rare.

## Case presentation

**Fig. 1:**
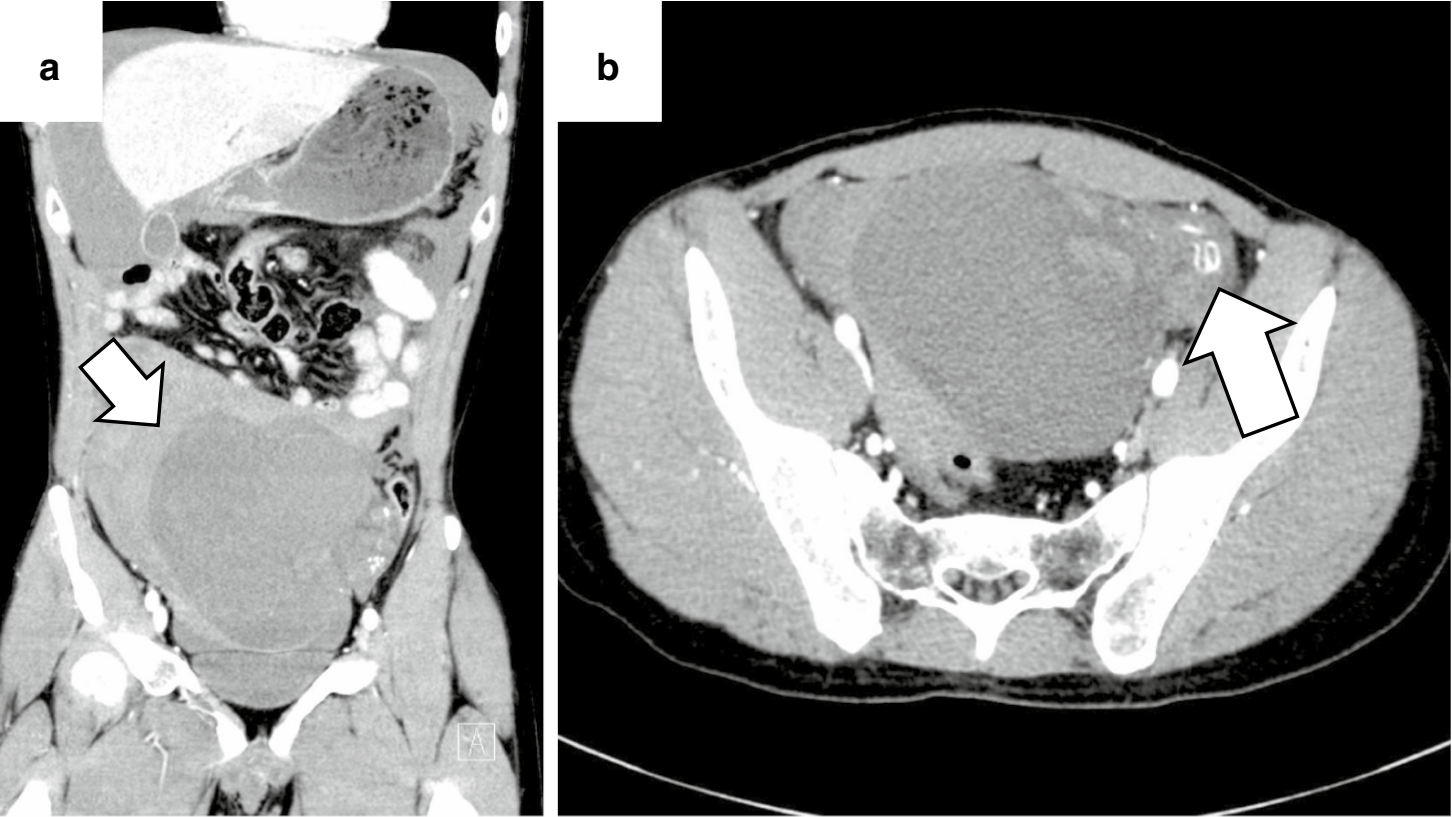
**a** A large volume of high-density ascites in the abdominal cavity and a huge mass in the pelvis were observed. **b** The contrast enhancement effect of the mass was poor, and blood vessels were observed inside the mass (arrow)

**Fig. 2:**
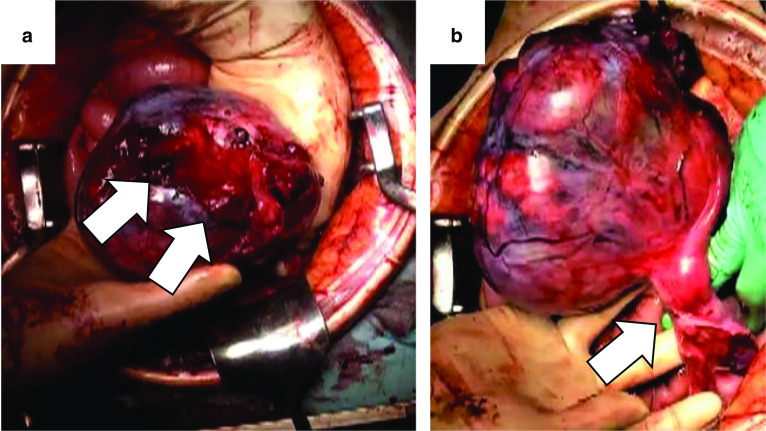
**a** The capsule of the mass was torn and caused bleeding (arrow). **b** The mass was connected by the feeding vessels of the left gonadal artery and vein only (arrow)

**Fig. 3:**
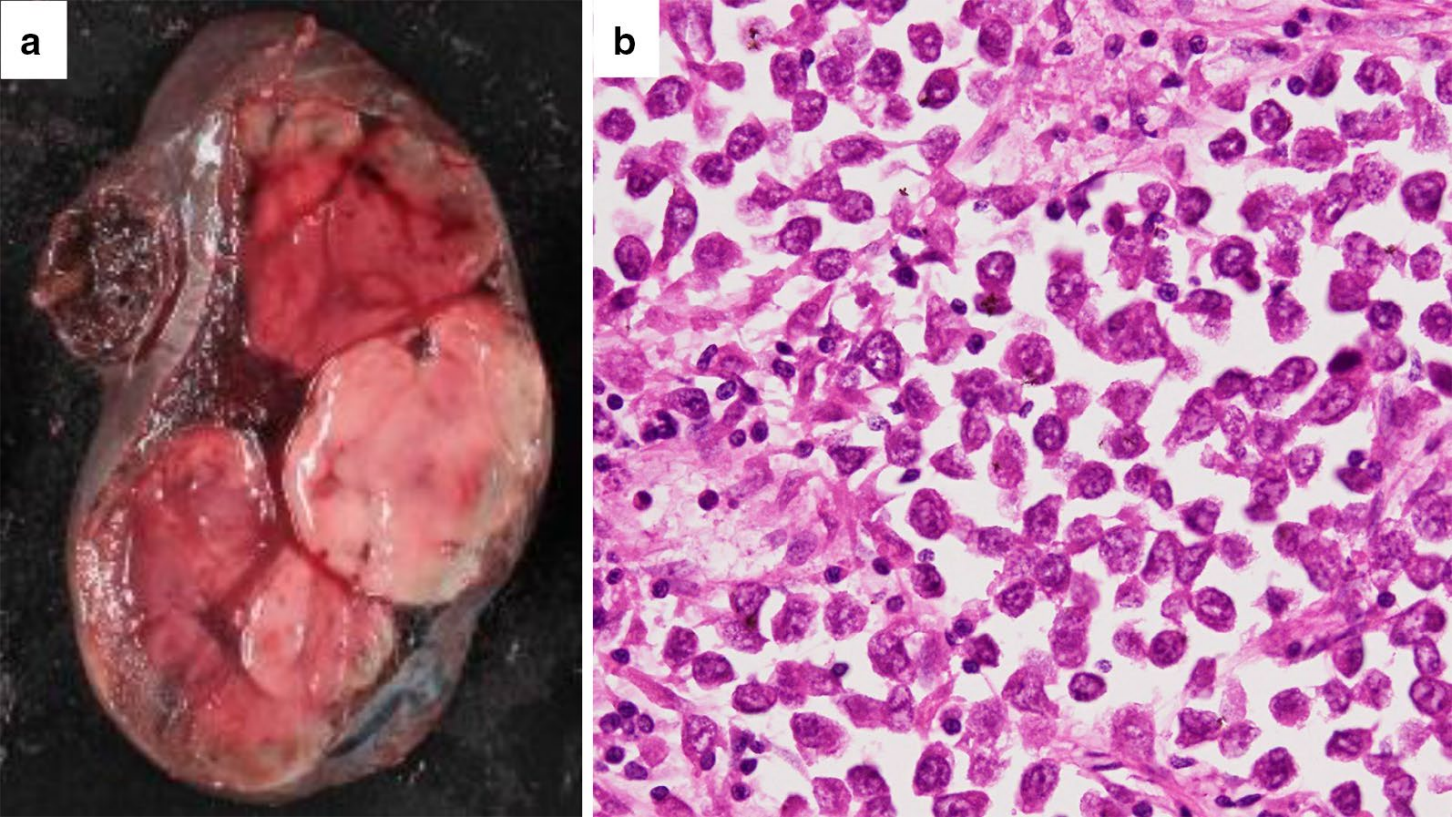
**a** Gross photograph of the mass cross-section. Solid tumor covered with a thin capsule showing internal necrosis and hemorrhage. **b** Tumor cells with large circles are arranged in sheets, with finely vacuolated cytoplasm, and nuclei with distinct nucleoli of the tumor cells are round. Infiltration of lymphoplasmacytic cells is observed (hematoxylin and eosin stain, 400×)

A 35-year-old married father of a newborn infant was transferred to the emergency department complaining of abdominal pain and an approximate 10-h history of vomiting. He reported a surgical history of left undescended testis in infancy. He and his parents had noticed that his left scrotum was still small after the surgery, but they had not paid attention to it and had not seen a doctor. He denied any other significant medical or surgical history, or a history of injury. On admission, he was conscious, pale, and had a tachypnea. His blood pressure was 80/55 mmHg, his pulse rate was 130 beats/min, and he was in shock. On clinical examination, he presented with abdominal distention and diffuse tenderness with guarding. His blood test results showed a low hemoglobin concentration (9.2 g/dl) and an elevated white blood cell count (21,200/mm^3^). Contrast-enhanced computed tomography (CT) revealed a large volume of high-density ascites and a large mass, with a major axis of approximately 15 cm in the pelvic area. The mass was poorly attenuated and contained blood vessels (Fig. 1). There was no evidence of suspected metastasis to other organs or lymph nodes, paracentesis confirmed bloody fluid. We diagnosed hemorrhagic shock due to rupture of the pelvic mass, which was suspected to be a gastrointestinal stromal tumor. He was infused with 2500 ml of extracellular fluid preoperatively. Blood pressure improved with infusion, but decreased when the administration rate was slowed. We decided to perform an emergency laparotomy. It took 110 min from the admission to the start of the surgery. On opening the abdomen, approximately 1500 ml of bloody fluid and a torn mass with active bleeding were encountered. The mass was easily elevated to the outside and was only connected by a pedicle containing the left gonadal artery and vein (Fig. 2). The pedicle was twisted approximately 180°. The left gonadal artery and vein were ligated and divided, and the mass was excised. The mass was 16 × 12 × 9 cm in size and weighed 840 g. The operating time was 77 min, and the total blood loss was 1647 ml. He received 4 units of RBCs and 2000 ml of fluid infusion during surgery. The post-operative course was uneventful, and he was discharged on post-operative day 6. The cut surface of the soft tumor was composed of solid nodules with areas of hemorrhagic necrosis surrounded with a thin capsule. Histologic examination revealed clear tumor cells arranged in sheets with large, rounded nuclei proliferation and lymphocyte infiltration. There was neither vascular nor capsular invasion. The patient was diagnosed with a seminoma arising in the undescended testis (Fig. 3). Intra-operative ascites cytology showed no malignancy. No distant metastasis or lymph node metastasis was found on laparotomy and pre-operative CT, and his pathological staging was stage IA (pT1N0M0). Post-operative palpation demonstrated an absence of the left testis, resembling an empty scrotum. He had a surgical scar on his left inguinal region, but no other surgical scars could be identified. After discharge, he was followed up at the urology department of our hospital. One year following surgery, lactate dehydrogenase and alpha-fetoprotein tumor markers were found to be within normal limits; however, the human chorionic gonadotropin level was elevated. CT results revealed retroperitoneal lymph node recurrence. After three cycles of chemotherapy with bleomycin, etoposide, and cisplatin (20 mg/m^2^ cisplatin on days 1–5, 100 mg/m^2^ etoposide on days 1–5, and 30 kU bleomycin on days 1, 8, and 15, repeated 21 days), he underwent retroperitoneal lymph node dissection by transperitoneal approach by open surgery. Histological examination of dissected lymph nodes showed fibrous scarring with no residual tumors. One year after the radical retroperitoneal lymph node dissection, he remains well without any signs or symptoms of tumor recurrence.

## Discussion

This patient presented with shock due to bleeding from an abdominal tumor, for which the treatment of choice is an emergency laparotomy irrespective of a pre-operative diagnosis of the tumor. Our patient had a history of surgery for a left-sided undescended testis in infancy; however, the medical records for surgery and post-operative follow-up were not available. The intra-abdominal bleeding was due to rupture of a seminoma arising from the remnant testis. Post-operative examination and palpation indicated a left empty scrotum. The potential cause of intra-abdominal remnant testis even after orchiopexy is considered to be due to post-operative testicular retraction [5] or intra-operative inadequate confirmation of the intraperitoneal testis [6]. Polyorchidism with more than three testes per individual has also been reported [7], and it is possible that only one testis was treated during orchiopexy. He only had a surgical scar on his left inguinal region, which suggested inadequate surgical exploration for the undescended testis. The fact that he and his family had noticed the empty scrotum even after the surgery for the undescended testes, but had not followed up, could also have been a problem. According to the "evaluation and treatment of cryptorchidism: AUA guidelines" published in 2014 [8], it is recommended that boys who have undergone treatment for undescended testes be taught how to perform testicular self-examination during adolescence and instructed to perform the examination monthly because the risk of testicular malignancies is still higher even after the reposition of the undescended testis.

The pathological staging of this case was stage I. For stage I testicular seminoma, radical inguinal orchiectomy is the first choice. Standard options after surgery include active surveillance, radiation therapy or 1–2 cycles of carboplatin [9]. In this case, active surveillance was performed postoperatively.

Malignant tumors arising from undescended testis have no characteristic symptoms; therefore, early determination can sometimes be challenging [10]. Enlarged intra-abdominal testicular tumors may cause urinary frequency due to bladder compression, bowel obstruction, torsion of the pedicle, and rupture with or without hemorrhage, according to tumor size [11].

A precise pre-operative diagnosis can offer reasonable or ideal surgical strategies for abdominal tumors. According to the condition of a patient with a ruptured abdominal tumor, interventional radiology may affect the post-operative course in terms of temporal hemostasis or reduction of blood inflow. Stabilization and recovery from the shock status generally allow for pre-operative therapies or less invasive surgery, including laparoscopic resection. A pre-operative diagnosis is critical to perform an appropriate urgent surgical procedure to control intra-abdominal hemorrhage immediately. For this patient, the necrotic and ruptured tumor had a thin distorted stalk containing the left gonadal artery and vein, which were connected to the inguinal region. Thus, torsion of the undescended testicular tumor’s pedicle was suspected immediately following the laparotomy. Our pre-operative diagnosis was not sufficiently precise, even on second look and review of the pre-operative CT. He was in shock on admission preoperatively, so we were unable to perform the full physical examination, including the scrotal examination. If we had noticed his empty scrotum before surgery, we would have suspected an intra-abdominal testicular tumor and first found and ligated the gonadal artery and vein, the feeding vessel of the mass, when we opened the abdomen.

Intra-abdominal hemorrhage from a seminoma arising from an undescended testis located in the abdominal cavity is a rare condition, with only five reports since 1970 of bleeding due to an intra-abdominal seminoma [12–16] (Table 1). According to these reports, one patient had a history of previous surgery for an undescended testis [13]. All five patients had undergone tumor resection, and distant or lymph node metastasis was not detected in pre-operative and/or intra-operative findings. Although acceptable post-operative short-term outcomes were described in all cases, the long-term results regarding tumor recurrence remain unclear. A collation of seminoma cases arising from an undescended testis and a comparison with an ordinal seminoma is warranted to delineate the biological nature and clinical effects of such seminomas on long-term outcomes.

**Table 1:** The literature review of cases of intraperitoneal hemorrhage from rupture of intra-abdominal testicular seminoma

References	Age (years)	Past surgery of undescended testis	Operation	Metastasis at initial diagnosis	Length of post-operative stay (days)	Recurrence
[12]	17	No	Tumor resection	No	7	No
[13]	38	Yes	Tumor resection	No	5	Not listed
[14]	28	No	Tumor resection	No	7	Not listed
[15]	30	No	Tumor resection	No	3	No
[16]	30	No	Tumor resection	No	Not listed	No
Current report	35	Yes	Tumor resection	No	6	Retroperitoneal lymph node

## Conclusion

We report a patient with an intra-abdominal testicular seminoma who was in hemorrhagic shock due to tumor rupture. An intra-abdominal seminoma may develop, even after previous orchiopexy surgery for undescended testes, and careful observation of an empty scrotum is advisable.

## Data Availability

The datasets supporting the conclusions of this article are included within the article and its additional files.

## Abbreviations

CT: Computed tomography
GIST: Gastrointestinal stromal tumor
